# Supplementary material for: Transcriptional profiling of human microglia reveals grey–white matter heterogeneity and multiple sclerosis-associated changes
Source: Nat Commun. 2019 Mar 13;10:1139. doi: 10.1038/s41467-019-08976-7 (PMC6416318; doi:10.1038/s41467-019-08976-7)
Supplement: Supplementary file 1 — Supplementary Information [file 41467_2019_8976_MOESM1_ESM.pdf]

Supplemental material

Transcriptional profiling of human microglia reveals grey–white matter heterogeneity and multiple sclerosis-associated changes

van der Poel et al.

Supplementary Table 1. Primer pairs used for RT-qPCR

Gene symbol	Forward sequence (5'–3')	Reverse sequence (5'–3')
ADGRG1	CTTCCAGCTTGTCGTCCTCTA	GCATGGACCAGTACCAGATGA
ADGRG1 (exon 15)	CTCTCCTAAGAGGTTCTCTCCA	CTACAACAGGCCAGCAATCTA
CD206	TGCAGAAGCAAACCAAACCTGTAA	CAGGCCTTAAGCCAACGAAACT
CD45	GCAGCTAGCAAGTGGTTTGTTC	AAACAGCATGCGTCCTTTCTC
CHI3L1	TTTGATGGGCTGGACCTTG	GCTGGGCTTCCTTTATAAATTCG
CHI3L1 (exon 10)	GGTCCACAACACACAGATT	AATCCCGAGTCTTACATTGC
CSF1R	GGCCTGCAAGGTTTTAACTG	GAGAGGGTGAAGGTGTGC
CX3CR1	TTGGCCTGGTGGGAAATTTGT	AGGAGGTAAATGTCGGTGACACT
EEF1A	AAGCTGGAAGATGGCCCTAAA	AAGCGACCCAAAGGTGGAT
EEPD1	TGACACTCCTGGAAAACAGC	TCCACTTGCGGATGTTGG
EEPD1 (exon 8)	GAACCCTTGGATTCCGGATAA	CCTTCTTGCTCCAGTCCTTT
GAPDH	TGCACCACCAACTGCTTAGC	GGCATGGACTGTGGTCATGA
GPR34	GCGAACCTGAACTTTAATGCAA	GTCGCAACCTTTCACCGTTT
LPL	ACTTGCCACCTCATTCCC	ACCCAACTCTCATACATTCCTG
LPL (exon 10)	CAGCCCTACCCTTGTTAGTTATT	ACGTTGGAGGATGTGCTATTT
P2RY12	TTCAAACCCTCCAGAATCAACAG	GTGCACAGACTGGTGTTACC
P2RY13	TATCCTCCCAAAGGTGACACTG	TACCAGCTGTACTATCCGAGTG
SLC2A5	CCGTGTCCATGTTTCCATTTG	CGCAGGCACGATAGAAAATATG
SLPI	CTGTGGAAGGCTCTGGAAAG	TGGCACTCAGGTTTCTTGTATC
TMEM119	CCACTCTCGCTCCATTCG	CAGCAACAGAAGGATGAGGA

Supplementary Table 2. Antibodies used for Western blot analysis

Antibody	Catalogus	Dilution	Company
Beta-actin	SC-47778	1:1,000	Santa Cruz
GPR56	358203	1:100	BioLegend
IkBα	4814	1:500	Cell Signaling
pIkBα	9246	1:500	Cell Signaling
p100/p52	05-361	1:500	Millipore

Supplementary Table 3. Antibodies and reagents used for flow cytometry

**Antibody/Reagent**	**Clone**	**Fluorochrome**	**Dilution**	**Company**
CD11b	ICRF44	PE	1:200	eBioscience
CD14	MϕP9	APC-H7	1:100	BD Biosciences
CD15	HI98	PerCP-Cy5.5	1:20	BD Biosciences
CD45	HI30	BB515	1:200	BD Biosciences
GPR56	CG4	PE-Cy7	1:100	BioLegend
Fixable Viability Dye eFluor™ 506	-	-	1:500	ThermoFisher

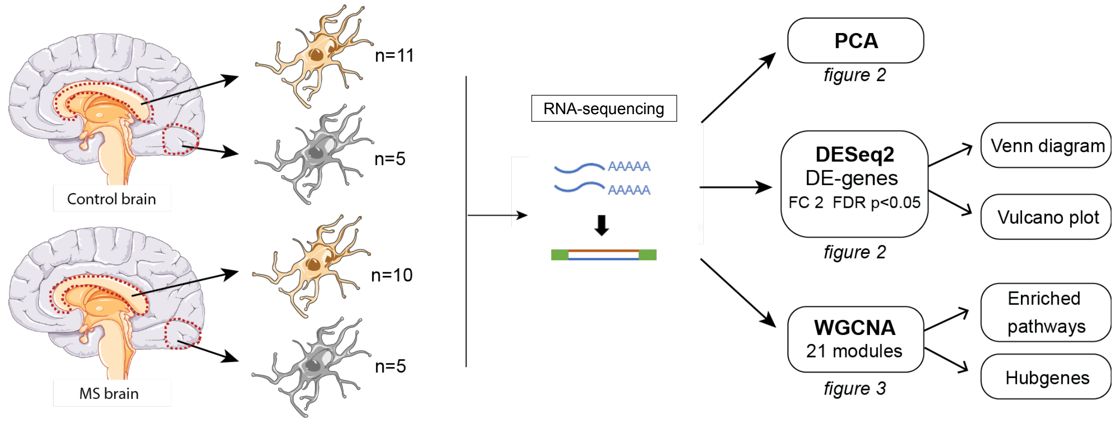

Supplementary Figure 1. Experimental workflow for RNA-sequencing analysis of human microglia. Microglia were isolated from control donors, GM (n=5) and WM (n=11) regions, and from MS donors, GM (n=5) and WM (n=10) regions. RNA-sequencing was performed and global gene expression was identified by PCA, differentially expressed genes were determined using DESeq2, and both Venn diagram and Vulcano plots were generated. WGCNA was performed to identify co-expression networks and shows 21 modules, with enriched pathway analysis and hub genes. Images of the brain and microglia were adapted from Servier Medical Art by Servier licensed under a Creative Commons Attribution 3.0 Unported License.

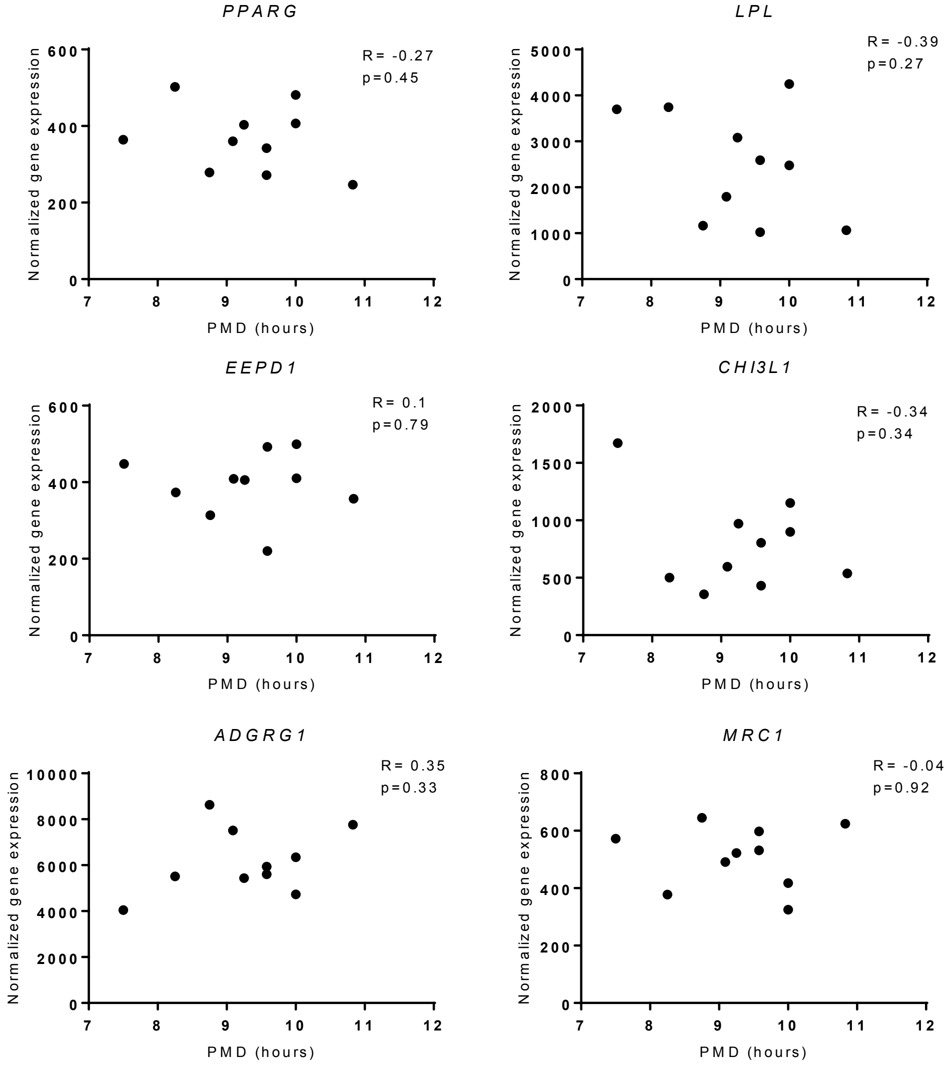

Supplementary Figure 2. Correlations between post-mortem delay and MS-related DE-genes. MS-related DE-genes show no significant correlation with post-mortem delay (PMD) for *PPARG*, *LPL*, *EEPD1*, *CHI3L1*, *ADGRG1* and *MRC1* in 10 MS donors. Pearson correlation test.

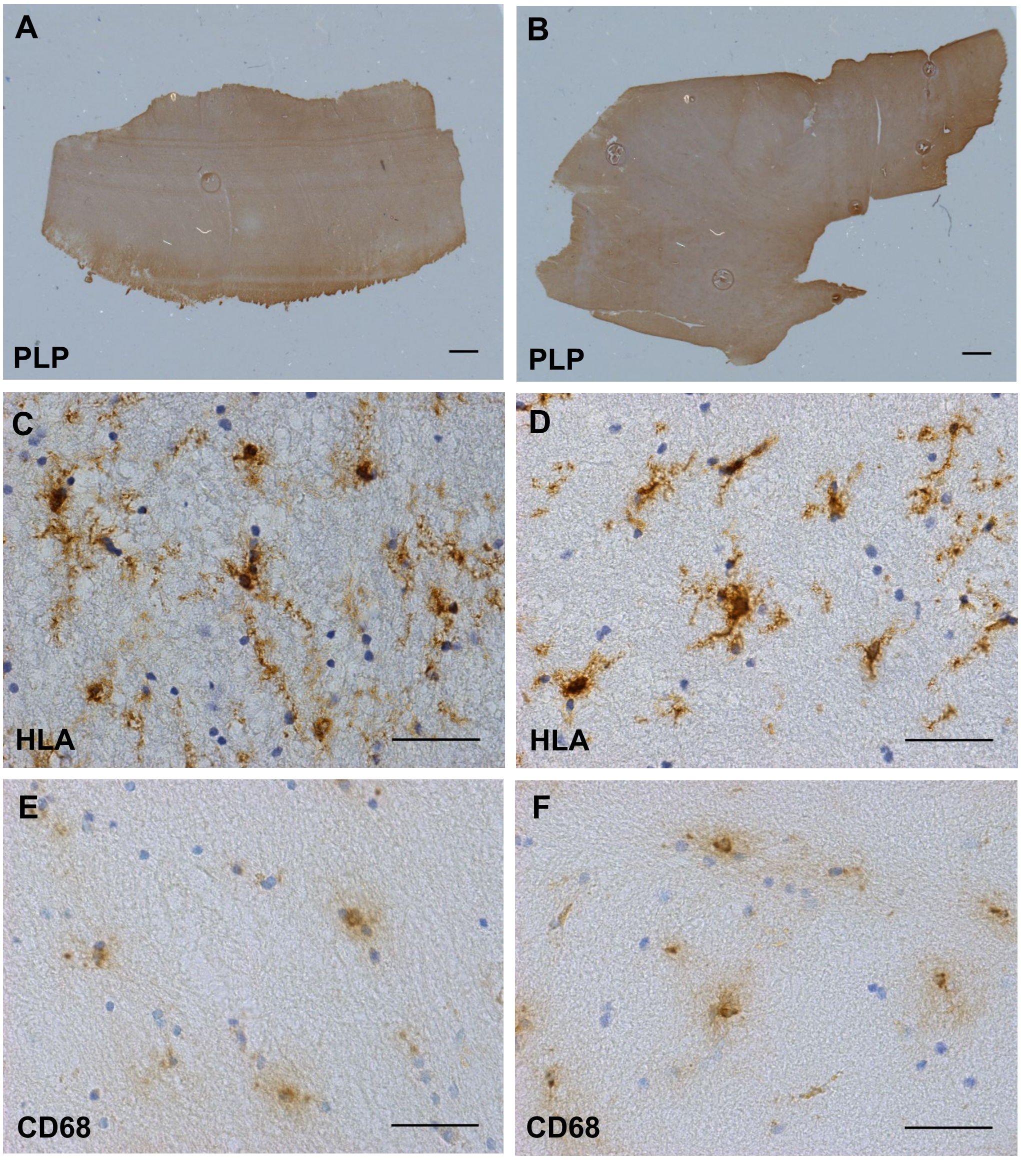

Supplementary Figure 3. Myelin integrity and microglia morphology in control and MS WM tissue used for RNA-sequencing. No signs of demyelination in WM tissue of MS **(A)** and control donors **(B)** based on myelin proteolipid protein (PLP) staining, scale bar 100 µm. Microglia show ramified morphology and no difference in expression of activation markers HLA-DR and CD68 in both MS **(C, E)** and control donors **(D, F)**, scale bar 50 µm. Shown are representative stainings of a MS (NBB number 12-043) and a control donor (NBB number 11-044).

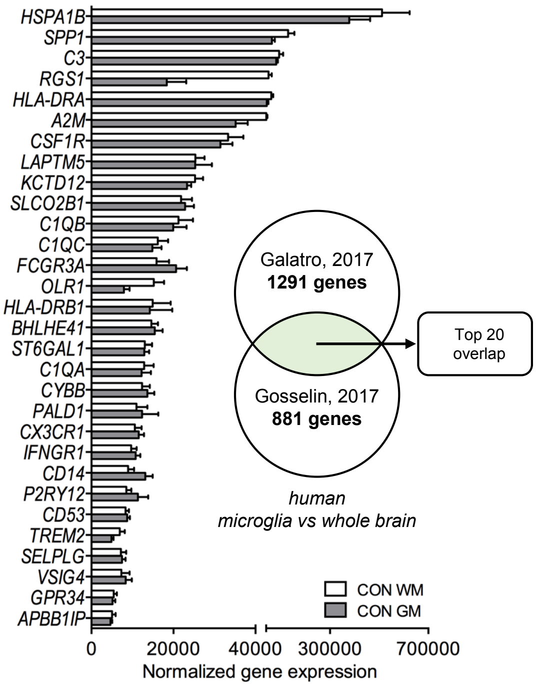

Supplementary Figure 4. Expression of human microglia signature genes in grey-white matter microglia. Expression of top 30 human microglia enriched genes, that overlap in two datasets[2,3] is conserved in GM and WM microglia in our dataset. Bars show mean with standard deviation.

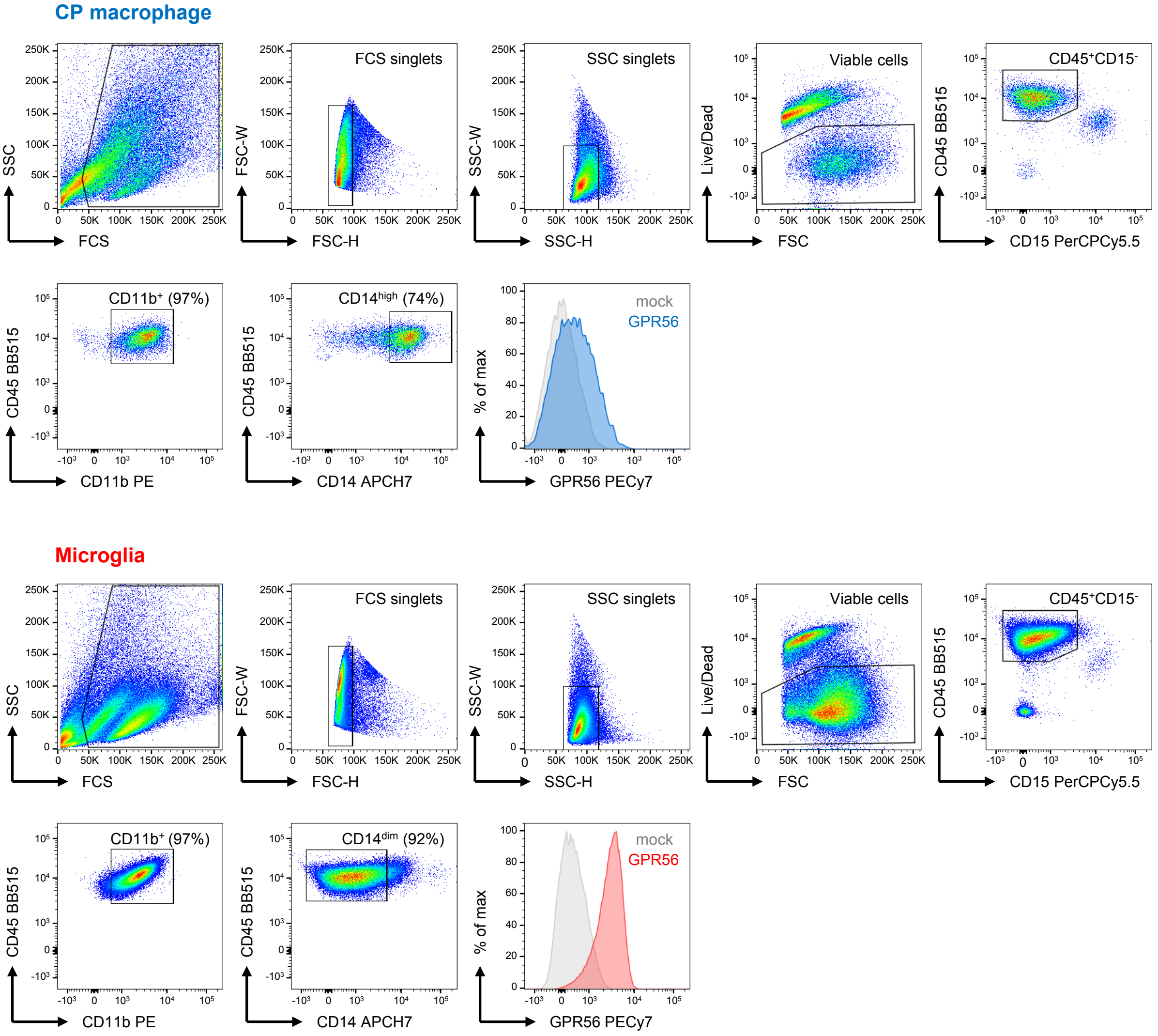

Supplementary Figure 5. Flow cytometry gating strategy for human microglia and macrophages. Representative dot plots showing the gating strategy used for Figure 1E with from left to right: cell gating by forward scatter (FCS) and sideward scatter (SSC), FCS-width/height duplet exclusion, SSC-width/height duplet exclusion, gating of viable cells, gating of $CD45^{+}CD15^{-}$ events, gating of $CD11b^{+}$ events, and gating of $CD14^{dim/high}$ events. Previous study shows high expression of CD14 by choroid plexus macrophages and $CD14^{dim}$ expression by microglia[4]. Representative histograms show GPR56 expression in $CD14^{high}$ CP macrophage and $CD14^{dim}$ microglia.

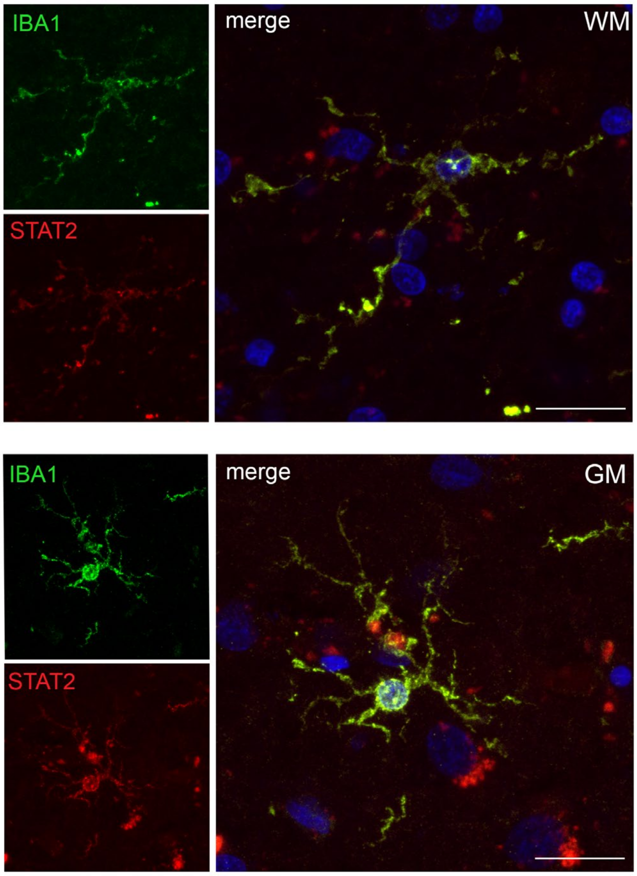

Supplementary Figure 6. STAT2 expression by microglia in grey and white matter control tissue. Immunohistochemical detection of the expression of STAT2 in WM and GM microglia in control brain tissue, confirmed in three donors. Scale bar is 20 µm.

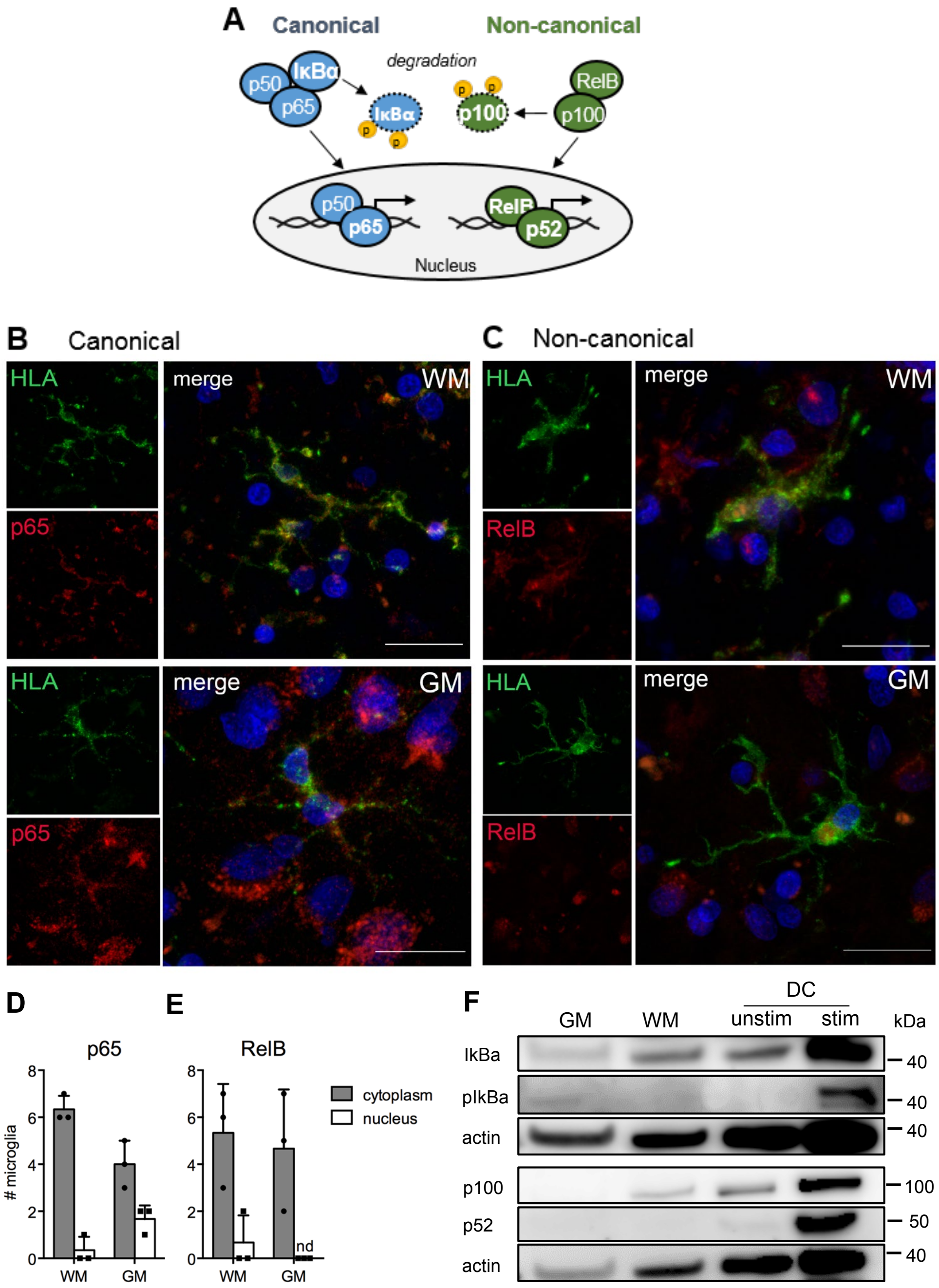

Supplementary Figure 7. Marked expression of NF-κB subunits in grey and white matter microglia. (A) Schematic overview of canonical and noncanonical NF-κB pathways. **(B-E)** Immunohistochemical detection of the expression of NF-κB subunits p65 (canonical) and RelB (noncanonical) shows no nuclear translocation in major percentage of HLA-positive GM and WM microglia in control tissue, confirmed in three donors. **(F)** Western blot analysis shows no activation of NF-κB pathways in GM and WM microglia, as measured by phosphorylation status of IkBα (canonical NF-κB signaling) or processing of p100 to p52 (active noncanonical NF-κB signaling), confirmed in three donors. Monocyte-derived dendritic cells (DC) stimulated with anti-CD40 was used as positive control for NF-κB activation**.** Nd = not detected, scale bar is 20 µm. Bars show mean with standard deviation.

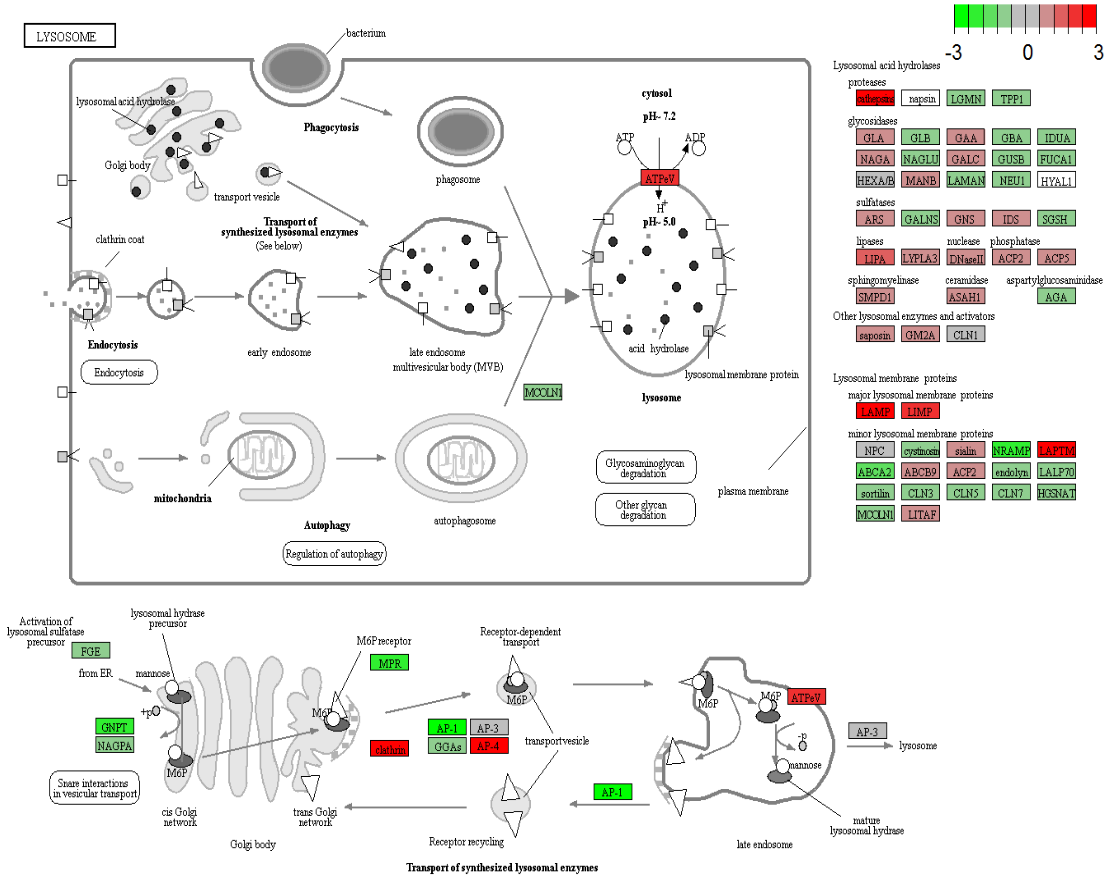

Supplementary Figure 8. KEGG pathway analysis identified lysosomal pathway in MS WM microglia. Co-expression network analysis identified module darkmagenta correlated with MS WM microglia, and shows 'lysosome' as enriched KEGG pathway within this module. Genes up- or downregulated are displayed in red or green, based on fold change, for the comparison MS WM *vs* control WM.

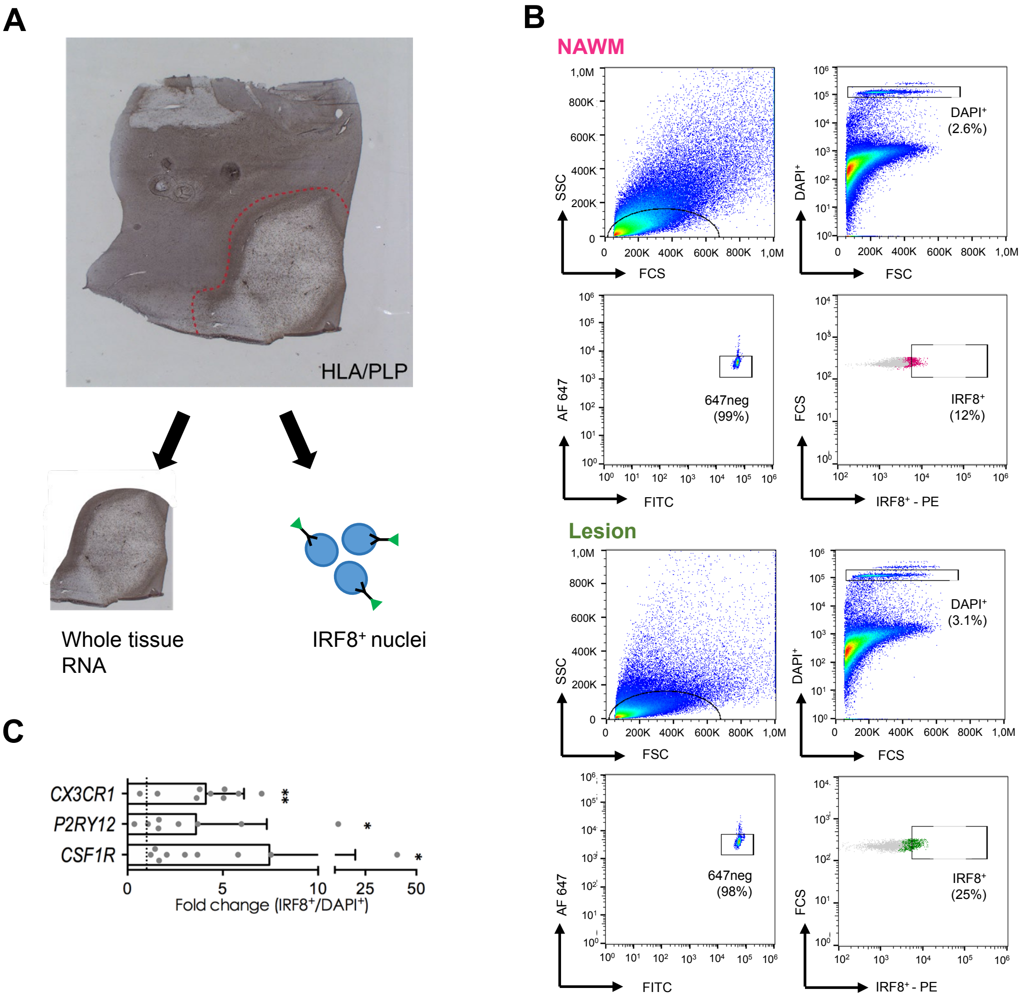

Supplementary Figure 9. Whole tissue RNA and IRF8$^+$ nuclear RNA from NAWM and active MS lesions for gene expression analysis.

(A) RNA was isolated from whole tissue and sorted IRF8$^+$ nuclei obtained from NAWM tissue (n=7) and dissected MS lesions (n=5). **(B)** Representative dot plots showing the gating strategy for sorting of IRF8$^+$ nuclei from NAWM or a MS lesion from left to right: small nuclei selection based on sideward scatter (SSC) and forward scatter (FSC), DAPI$^+$ nuclei selection, nuclei negative for autofluorescence signal in 647 channel, and selection of IRF8$^+$ nuclei compared to isotype control. **(C)** IRF8$^+$ nuclei from WM tissue (n=9) show enriched expression of microglia genes *CX3CR1*, *P2RY12*, and *CSF1R* compared to complete DAPI$^+$ fraction (n=9). Wilcoxon test or one-tailed paired t-test: * $p< 0.05$, ** $p<0.01$. Bars show mean with standard error.

References

1. Luchetti, S. *et al.* Progressive multiple sclerosis patients show substantial lesion activity that correlates with clinical disease severity and sex: a retrospective autopsy cohort analysis. *Acta Neuropathol.* **135,** 511–528 (2018).

2. Gosselin, D. *et al.* An environment-dependent transcriptional network specifies human microglia identity. *Science.* **3222,** 33–35 (2017).

3. Galatro, T. F. *et al.* Transcriptomic analysis of purified human cortical microglia reveals age-associated changes. *Nat. Neurosci.* **20,** 1162–1171 (2017).

4. Melief, J. *et al.* Microglia in normal appearing white matter of multiple sclerosis are alerted but immunosuppressed. *Glia* **61,** 1848–1861 (2013).
