# Supplementary material for: Transcriptional profiling of human microglia reveals grey–white matter heterogeneity and multiple sclerosis-associated changes
Source: Nat Commun. 2019 Mar 13;10:1139. doi: 10.1038/s41467-019-08976-7 (PMC6416318; doi:10.1038/s41467-019-08976-7)
Supplement: Supplementary file 3 — Description of Additional Supplementary Information [file 41467_2019_8976_MOESM3_ESM.pdf]

Description of Additional Supplementary Files

File Name: Supplementary Data 1
Description: **Donor characteristics.** Explanation of abbreviations used in the table: Active lesion load = number of active MS lesions/total number of MS lesions in all tissue blocks dissected[1]; AD = Alzheimer's disease; Age = age at death (years); CC = corpus callosum; CON = non-neurological control; CP = choroid plexus; CVA = cerebrovascular accident; Disease duration is time between MS diagnosis until death in years; F = female; FACS = flow cytometry; IHC = immunohistochemistry; LBD = Lewy body disease; LBV = Lewy body variant; M = male; MS PP = primary progressive MS; MS SP = secondary progressive MS; NAWM = normal-appearing white matter; NBB no. = donor registration number of the Netherlands Brain Bank; OC = occipital cortex; Peripheral inflammation = peripheral inflammation at time of death; PMD = post-mortem delay (h:min); PD = Parkinson's disease; PSP = Progressive supranuclear palsy; RT-qPCR = quantitative reverse transcription polymerase chain reaction; RIN = RNA integrity number; RNA-seq = RNA sequencing; RR = relapsing remitting; SWM = subcortical white matter; U = unknown; UTI= Urinary tract infection; WB = Western blot analysis; WM = white matter; *MS diagnosis was not yet confirmed by neuropathologist.

File Name: Supplementary Data 2
Description: **DE genes and GO-term analysis for grey and white matter microglia in control and MS donors.** DE genes were defined among grey and white matter microglia for both control and MS donors, based on a fold change of >2 or <-2 and adjusted p-value <0.05. Normalized mean values are shown. GO term analysis was performed for all DE-genes, based on adjusted p-values and gene names associated with each GO term are provided.

File Name: Supplementary Data 3
Description: **KEGG pathways identified by gene set enrichment analysis.** Gene set enrichment analysis identified enriched KEGG pathways for all 21 modules, that were defined by weighted gene co-expression analysis. Enriched pathways are shown with adjusted p-values and for each pathway gene names are provided.

File Name: Supplementary Data 4
Description: **Hallmark pathways identified by gene set enrichment analysis.** Gene set enrichment analysis identified enriched Hallmark pathways for all 21 modules that were defined by weighted gene co-expression analysis. Enriched pathways are shown with adjusted p-values and for each pathway gene names are provided.

File Name: Supplementary Data 5
Description: **GO pathways identified by gene set enrichment analysis.** Gene set enrichment analysis identified enriched GO pathways for all 21 modules that were defined by weighted gene co-expression analysis. Enriched pathways are shown with adjusted p-values and for each pathway gene names are provided.

File Name: Supplementary Data 6
Description: **Disease pathways identified by gene set enrichment analysis.** Gene set enrichment analysis identified enriched disease pathways for all 21 modules that were

defined by weighted gene co-expression analysis. Enriched pathways are shown with adjusted p-values and for each pathway gene names are provided.

File Name: Supplementary Data 7
Description: **Hubgenes identified by connectivity analysis.** Connectivity analysis identified hubgenes with highest intramodular connectivity for all 21 modules that were defined by weighted gene co-expression analysis.

File Name: Supplementary Data 8
Description: **Uncropped Western blot showing GPR56 receptor in white matter microglia.** Uncropped Western blot showing expression of GPR56 (60 kDa) in white matter microglia and Natural Killer (NK)-92 cells expressing vector control or GPR56 as negative or positive control. Related to Figure 1F.

File Name: Supplementary Data 9
Description: **Uncropped Western blots showing NF-κB subunits in grey and white matter control microglia.** Uncropped Western blots showing expression of **(A)** IκBα (39 kDa), **(B)** pIκBα (39 kDa), **(C)** p100 (100 kDa), and **(D)** p52 (52 kDa) in both grey and white matter microglia isolated from three control donors. Actin was used as loading control and monocyte-derived dendritic cells (DC) stimulated with CD40 antibody was used as positive control for NF-κB activation. Related to Supplementary Figure 7.
