# Supplementary figures and images for: Transcriptional profiling of human microglia reveals grey–white matter heterogeneity and multiple sclerosis-associated changes
Source: Nat Commun. 2019 Mar 13;10:1139. doi: 10.1038/s41467-019-08976-7 (PMC6416318; doi:10.1038/s41467-019-08976-7)

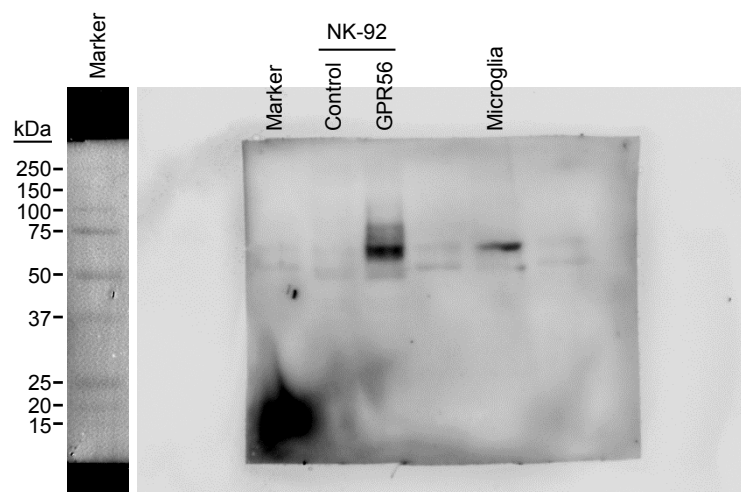

Supplement: Supplementary file 11 — Supplementary Data 8 [file 41467_2019_8976_MOESM11_ESM.pdf]

**A**

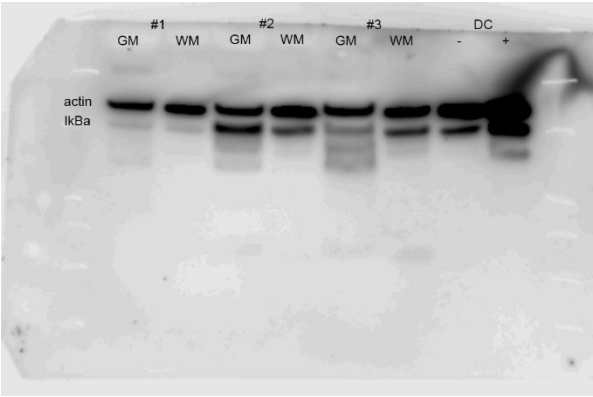

**B**

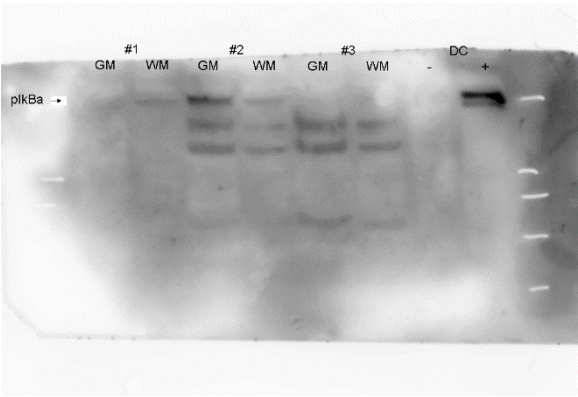

**C**

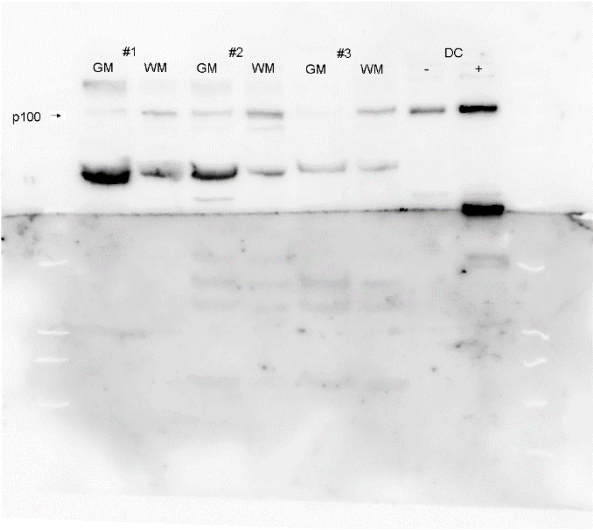

**D**

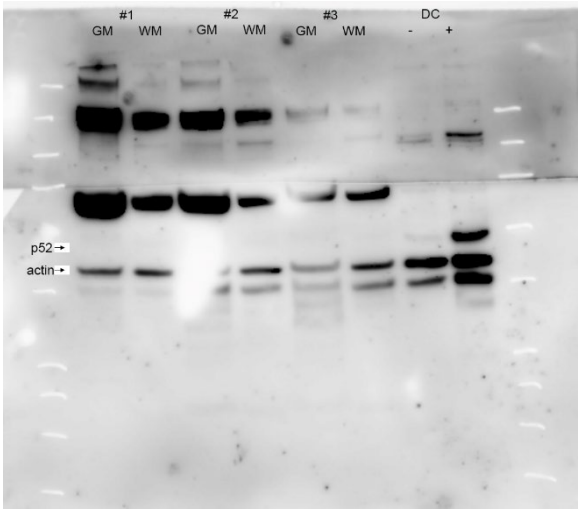

Supplement: Supplementary file 12 — Supplementary Data 9 [file 41467_2019_8976_MOESM12_ESM.pdf]
