# Supplementary material for: Transcriptional profiling of human microglia reveals grey–white matter heterogeneity and multiple sclerosis-associated changes
Source: Nat Commun. 2019 Mar 13;10:1139. doi: 10.1038/s41467-019-08976-7 (PMC6416318; doi:10.1038/s41467-019-08976-7)
Supplement: Supplementary file 13 — Reporting Summary [file 41467_2019_8976_MOESM13_ESM.pdf]

nature research

Corresponding author(s): Dr. I. Huitinga
Dr. J. Hamann

Last updated by author(s): 29-01-2019

Reporting Summary

Nature Research wishes to improve the reproducibility of the work that we publish. This form provides structure for consistency and transparency in reporting. For further information on Nature Research policies, see Authors & Referees and the Editorial Policy Checklist.

Statistics

For all statistical analyses, confirm that the following items are present in the figure legend, table legend, main text, or Methods section.

n/a	Confirmed	
☐	☒	The exact sample size (*n*) for each experimental group/condition, given as a discrete number and unit of measurement
☐	☒	A statement on whether measurements were taken from distinct samples or whether the same sample was measured repeatedly
☐	☒	The statistical test(s) used AND whether they are one- or two-sided<br>*Only common tests should be described solely by name; describe more complex techniques in the Methods section.*
☐	☒	A description of all covariates tested
☐	☒	A description of any assumptions or corrections, such as tests of normality and adjustment for multiple comparisons
☐	☒	A full description of the statistical parameters including central tendency (e.g. means) or other basic estimates (e.g. regression coefficient) AND variation (e.g. standard deviation) or associated estimates of uncertainty (e.g. confidence intervals)
☐	☒	For null hypothesis testing, the test statistic (e.g. *F*, *t*, *r*) with confidence intervals, effect sizes, degrees of freedom and *P* value noted<br>*Give P values as exact values whenever suitable.*
☒	☐	For Bayesian analysis, information on the choice of priors and Markov chain Monte Carlo settings
☒	☐	For hierarchical and complex designs, identification of the appropriate level for tests and full reporting of outcomes
☒	☐	Estimates of effect sizes (e.g. Cohen's *d*, Pearson's *r*), indicating how they were calculated

Our web collection on statistics for biologists contains articles on many of the points above.

Software and code

Policy information about availability of computer code

Data collection	Flow cytometry software BD FACS Diva version 8.1<br>Westerrn blots were visualized using a luminescent image analyzer, with Image Quant LAS 4000 mini, version 1.3<br>Confocal SP8 microscopy software Leica Application Suite X<br>RNAsequencing software NextSeq control v1/4/8
Data analysis	Flow cytometry data were analyzed using FlowJo software, version 10.1.<br>Confocal image analysis was performed with Fiji plugin for ImageJ1 software77.<br>Software used to analyse the RNA-sequencing data is described in the Methods section at page 18-19.

For manuscripts utilizing custom algorithms or software that are central to the research but not yet described in published literature, software must be made available to editors/reviewers. We strongly encourage code deposition in a community repository (e.g. GitHub). See the Nature Research guidelines for submitting code & software for further information.

Data

Policy information about availability of data

All manuscripts must include a data availability statement. This statement should provide the following information, where applicable:

- Accession codes, unique identifiers, or web links for publicly available datasets
- A list of figures that have associated raw data
- A description of any restrictions on data availability

The RNA-sequencing dataset is available online in the Gene Expression Omnibus (GEO) database (https://www.ncbi.nlm.nih.gov). The GEO accession number is GSE111972.

Field-specific reporting

Please select the one below that is the best fit for your research. If you are not sure, read the appropriate sections before making your selection.

☒ Life sciences ☐ Behavioural & social sciences ☐ Ecological, evolutionary & environmental sciences

For a reference copy of the document with all sections, see nature.com/documents/nr-reporting-summary-flat.pdf

Life sciences study design

All studies must disclose on these points even when the disclosure is negative.

Sample size	Sample size was not statistically predetermined due to limited sample availability.
Data exclusions	The RNA samples that did not meet the quality criteria for RNAsequencing, measured with the Fragment Analyzer, were excluded.
Replication	Microglial RNA and protein expression was defined in at least 3 different brain donors.
Randomization	Donor samples were not randomly allocated to a experimental group. Depending on region and diagnosis, donor samples were allocated to GM/WM and CON/MS group. Donors were matched for age, gender and RNA integrity values.
Blinding	Investigators were not blinded to group allocation during data analysis, but data analysis was performed unbiased using principal component analysis and weigthed gene co-expression network analysis.

Reporting for specific materials, systems and methods

We require information from authors about some types of materials, experimental systems and methods used in many studies. Here, indicate whether each material, system or method listed is relevant to your study. If you are not sure if a list item applies to your research, read the appropriate section before selecting a response.

Materials & experimental systems

n/a	Involved in the study	
☐	☒	Antibodies
☒	☐	Eukaryotic cell lines
☒	☐	Palaeontology
☒	☐	Animals and other organisms
☐	☒	Human research participants
☒	☐	Clinical data

Methods

n/a	Involved in the study	
☒	☐	ChIP-seq
☐	☒	Flow cytometry
☒	☐	MRI-based neuroimaging

Antibodies

Antibodies used	Antibodies used for Western blot analysis and flow cytometry are described in Supplementary Tables 3-4 and antibodies used for immunohistochemistry are described in the Methods section. For each antibody, the clone or catalogus number, fluorochrome, dilution and company is provided.
Validation	Antibodies for flow cytometry were titrated and validated using PBMCs, THP1 cells or primary human microglia.<br>Antibodies for Western blot analysis were validated using monocyte-derived dendritic cells stimulated with CD40 antibody.<br>Antibodies for immunohistochemistry were validated in previous studies (Luchetti et al, Acta Neuropathol, 2018; Tas et al, Eur J Immunol, 2005).<br>For all experiments, negative controls were included (complete staining procedure without primary antibody).

Human research participants

Policy information about studies involving human research participants

Population characteristics	See Table 1 for a description of all donor characteristics.
Recruitment	Human brain tissue was provided by the Netherlands Brain Bank (https://www.hersenbank.nl/). Brain donors signed up for the donor program of the Netherlands Brain Bank and gave consent for the use of brain tissue and clinical information for research purposes.
Ethics oversight	The NBB has obtained approval from the medical ethics committee of VU University medical center (VUmc, Amsterdam, The Netherlands). The ethics statement is available at www.brainbank.nl/media/uploads/file/Ethical-declaration.pdf.

Note that full information on the approval of the study protocol must also be provided in the manuscript.

Flow Cytometry

Plots

Confirm that:

☒ The axis labels state the marker and fluorochrome used (e.g. CD4-FITC).

☐ The axis scales are clearly visible. Include numbers along axes only for bottom left plot of group (a 'group' is an analysis of identical markers).

☒ All plots are contour plots with outliers or pseudocolor plots.

☐ A numerical value for number of cells or percentage (with statistics) is provided.

Methodology

Sample preparation	See Methods section 'Flow-cytometric analysis' on pages 21-22.
Instrument	3-laser BD FACSCanto IITM machine .
Software	FlowJo software version 10.1 .
Cell population abundance	Percentage microglia (97%) and macrophages (97%) was based on CD45+CD11b+CD15- expression (see Supplementary Figure 5). Percentage IRF8+ nuclei was based on DAPI+ and IRF8+ expression, compared to isotype control for IRF8: 12% IRF8+ in NAWM tissue and 25% IRF8+ in lesion tissue (see Supplementary Figure 9).
Gating strategy	For flow-cytometric analysis of GPR56 expression in microglia and macrophages, see Supplementary Figure 5.<br>For IRF8+ nuclei sorting, see Supplementary Figure 9.

☒ Tick this box to confirm that a figure exemplifying the gating strategy is provided in the Supplementary Information.
